# Diagnosis and Management of Pembrolizumab-Associated Pericardial Effusion in a Non-small Cell Lung Cancer Patient

**DOI:** 10.7759/cureus.37556

**Published:** 2023-04-14

**Authors:** James Pollock, Elquis Castillo

**Affiliations:** 1 Medical School, Edward Via College of Osteopathic Medicine, Auburn, USA; 2 Hematology and Oncology, Hematology and Oncology Associates of Alabama, Gadsden, USA

**Keywords:** pericardial tamponade, non-small cell lung cancer, immune checkpoint inhibitors, future management, pericardial window

## Abstract

The advent of immune checkpoint inhibitors (ICIs) in the field of oncology has improved the outcome response rate for a variety of neoplastic pathologies with improved cellular specificity that lacks the traditional adverse effects associated with chemotherapy. However, ICIs are not without adverse associations, and a growing concern for modern clinicians is the balancing of interests that most occur to minimize these adverse effects while also improving patients’ conditions from an oncologic perspective. This case presents a 69-year-old man who developed multiple episodes of significant pericardial effusion while receiving infusions of pembrolizumab for stage III-A adenocarcinoma for which he underwent a pericardiostomy procedure. Given the positive response of this immunotherapy on disease progression, the decision was made to continue the administration of pembrolizumab following the pericardiostomy with the plan of using serial echocardiography studies to monitor for the presence of clinically significant pericardial effusion in the future. In this way, the patient will still be able to receive optimal treatment for his advanced cancer while preserving adequate cardiac function.

## Introduction

Humanized monoclonal antibodies against programmed cell death protein 1 (PD-1), such as pembrolizumab and nivolumab, have significantly improved the ability to induce a therapeutic response to advanced non-small cell lung cancers (NSCLCs). In fact, the overall response rate for pembrolizumab specifically has been estimated to be 24.8% in previously untreated patients and 18% in those receiving prior cancer treatment [1]. This response rate remains elevated even with enhanced tumor expression of programmed cell death ligand 1 (PD-L1) [2]. A caveat to the use of anti-PD-1 agents and other immune checkpoint inhibitors (ICIs), however, is the incidence of immune-related adverse events (irAEs) that are unique to each agent and distinct from historical chemotherapy toxicities. For example, anti-PD-1 agents have been shown to be associated with complicating pneumonitis, especially in NSCLC patients with a history of pulmonary fibrosis [3]. Pembrolizumab has been shown to be a rare cause of pericardial effusion with the potential for acute pericardial tamponade, despite the fact that irAEs involving the cardiovascular system are generally rare with an estimated incidence of less than 0.6% [4,5]. Given the therapeutic potential for these agents in patients with advanced-stage cancer, an ideal treatment plan would theoretically involve their continued use with the clinical management of complicating irAEs [6].

The following case presents a 69-year-old male patient diagnosed with stage III-A adenocarcinoma of the lung (T2N2M0) with a history of chemotherapy and radiation who developed pericardial effusion secondary to pembrolizumab immunotherapy. While essential to discuss this rare irAE, a series of considerations pertaining to the management of pericardial effusion in the setting of anti-tumor immunotherapy are also presented. As always, these considerations should account for patient preferences.

## Case presentation

**Figure 1:**
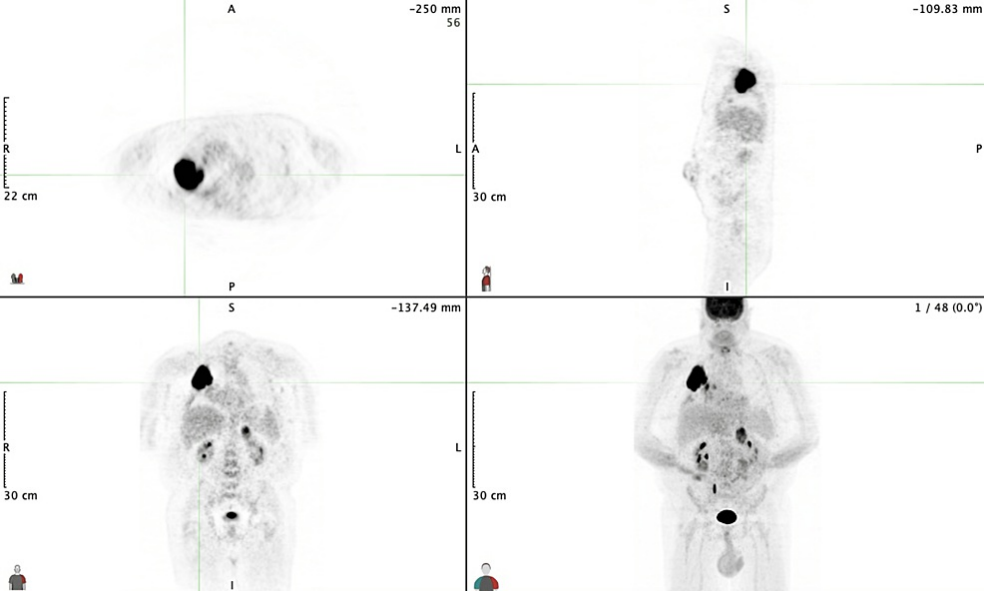
Surveillance positron emission tomography scan demonstrating mass-like uptake in the right lower lung lobe.

A 69-year-old Caucasian male with a history of previously treated lung cancer was referred to the hematology and oncology clinic to discuss his need to restart pembrolizumab immunotherapy for stage III-A adenocarcinoma of the right lung (T2N2M0). He had received multiple cycles of chemotherapy and radiation for adenocarcinoma six years ago and was declared free of disease following a negative positron emission tomography (PET) scan two years later. His current disease state was first noted on a surveillance PET scan three years ago with increased, mass-like uptake in the right lower lung lobe (Figure 1). CT-guided biopsy was positive for poorly differentiated adenocarcinoma, and PD-L1 combined positive score was 70%, consistent with a highly positive result. The decision was then made to initiate pembrolizumab monotherapy.

Despite one year of favorable disease response, the patient was taken off pembrolizumab one month ago because he was hospitalized twice within a one-month time period for diagnoses of pneumonia and pericardial effusion. The decision was made to undergo pericardiostomy following these inpatient stays, and he has not displayed symptoms of effusion relapse since the procedure. Because the immunotherapy had previously treated this patient’s adenocarcinoma with an adequate response, pembrolizumab was re-initiated with routine follow-up scheduled via echocardiogram to monitor for pericardial effusions that may be resistant to the pericardiostomy procedure. Additionally, routine oncology visits were scheduled to sufficiently monitor for adenocarcinoma disease progression.

## Discussion

This patient presents a difficult case because his interests as a cancer patient must be balanced with his need for sustained cardiac output via a reduction in the presence of pericardial effusion. Pericardiocentesis is typically considered the first-line treatment for pericardial effusion, but this should only be considered in cases of moderate-to-large effusions [7,8]. In this patient, the volume of pericardial effusion with which he presented to the hospital was enough to warrant intervention on two separate occasions, leading to the decision to hold pembrolizumab as it was the likely cause. From an oncology perspective, however, this decision meant jeopardizing the patient’s adenocarcinoma prognosis moving forward. A balance in interests was met via the pericardiostomy procedure, which allows for adequate drainage of pericardial effusions while still allowing for pembrolizumab infusions. This technique is preferred when there is a concern for recurrent effusion in a long-term care setting, as it allows for the drainage of fluid into either the mediastinum or thoracic cavity without the need for multiple pericardiocentesis procedures [9]. It is this element of discussion and compromise that makes this case report unique from other cases of pembrolizumab-associated pericardial effusion wherein cancer treatment was halted without an attempt at a pericardiostomy middle ground [5].

The use of serial echocardiography in this patient will be useful as he continues pembrolizumab infusions because it will allow for the comparison of future studies to a known, healthy baseline of cardiac function and effusion volume. Serial echocardiography has long been used to quantify ventricular performance in patients pre- and post-intervention, and these measurements can be acquired with respectable precision and accuracy [10]. Therefore, should pericardial effusion return following continued pembrolizumab, it will likely be detected without jeopardizing the patient’s health status. The detection of significant pericardial effusion in the future, despite undergoing pericardiostomy, would likely merit the permanent discontinuation of pembrolizumab for this patient.

## Conclusions

While pembrolizumab has been effective in the treatment of NSCLC, it is important to consider the adverse effects that may beget patients who are receiving drug infusions. Pericardiostomy remains a therapeutic treatment option for those who have experienced repeated episodes of pericardial effusion while taking pembrolizumab with a positive treatment response. Clinicians ought to maintain an understanding of the benefits of pericardiostomy in treating cancer patients with ICIs in future medical practice.
